# Predictors of COVID-19 Vaccine Acceptance among Healthcare Workers in Nigeria

**DOI:** 10.3390/vaccines10101645

**Published:** 2022-09-30

**Authors:** Victory Chizaram Nnaemeka, Reuben Ogba Onwe, Adaku Lydia Ekwedike, Oluwakemi Elizabeth Oyedele, Thomas Sambo Tsiterimam, Ogbole Emmanuel Ochepo, Nkiru Nenye Nwokoye, Anthony Chibuogwu Ike

**Affiliations:** 1 Department of Microbiology, Faculty of Biological Sciences, University of Nigeria, Nsukka 410001, Nigeria; 2 Applied Bioscience and Biotechnology, Faculty of Natural Sciences, South Kensington Campus, Imperial College London, London SW7 2AZ, UK; 3 Biomedical Sciences, College of Graduate Studies, SUNY Upstate Medical University, Syracuse, NY 13210, USA; 4 Cell Biology and Genetics, Faculty of Science, University of Lagos, Lagos 101017, Nigeria; 5 Department of Obstetrics and Gynaecology, University of Abuja Teaching Hospital, Abuja 902101, Nigeria; 6 Department of Biochemistry, School of Life Science, Federal University of Technology Minna, Minna 920101, Nigeria; 7 KNCV TB Foundation, Abuja 900211, Nigeria

**Keywords:** COVID-19 vaccine, acceptance, healthcare workers, knowledge, risk perception, Nigeria

## Abstract

Healthcare workers (HCWs) are regarded as role models regarding health-related issues, including vaccination. Therefore, it is essential to identify the predictors for COVID-19 vaccine acceptance among them. A cross-sectional study to assess the risk perception, attitudes and knowledge of HCWs toward COVID-19 vaccination was carried out. A total of 710 responses were received between September 2021 and March 2022, from HCWs in the Northern, Western and Eastern regions of Nigeria. Cross tabulations were performed to determine statistical relations between sociodemographic variables, knowledge, attitudes and risk perceptions concerning COVID-19 vaccine acceptance. Multinomial logistic regression analysis was performed to determine the predictive variables for COVID-19 vaccine acceptance. Statistical analyses were performed and P-values less than 0.05 were considered statistically significant at a CI of 95%. Results showed that 59.3% of the participants were amenable to COVID-19 vaccines. Multinomial regression analysis identified 14 variables at α < 0.05 as predictors for vaccine acceptance. Male HCWs were 2.8 times more likely to accept the vaccine than their female counterparts. HCWs that were knowledgeable of the different kinds of vaccines, were willing to recommend the vaccines to their patients, believed that the timing of COVID-19 vaccination was appropriate and had recent vaccination history within three years were 1.6, 24.9, 4.4 and 3.1 times more likely to take COVID-19 vaccine than those not sure. The study found a relatively high trust (51.3%) in the Nigerian Center for Disease Control (NCDC) for information regarding COVID-19 vaccines. Therefore, the NDCD should disseminate more robust insights regarding the safety profiles of various COVID-19 vaccines.

## 1. Introduction

Six vaccines (Pfizer–BioNTech, Moderna, Janssen (J & J), AstraZeneca–Oxford, Sinopharm and Sinovac (CoronaVac)) with different storage distribution logistics, ingredients and side effects received emergency use authorization by the United States Center for Disease Control on 6 August 2021 [1]. These vaccines have received different levels of acceptance in different regions of the world, commonly referred to as vaccine acceptance or hesitancy. Vaccine acceptance or hesitancy is a complex phenomenon caused by a plethora of factors that go beyond safety concerns [2]. Vaccine-hesitant individuals have been described as a heterogeneous population amid a spectrum that runs from complete acceptors to complete refusers [3]. These “hesitant” individuals may refuse some vaccines but agree to others, reject vaccines or are unsure of accepting vaccines [4].

The emergence of efficacious vaccination is essential to mitigating the high morbidity and mortality rate of COVID-19, as immunization is adequate to a varying extent in reducing the severity of complications [5,6], leading to reduced hospitalization and mortality [7,8]. COVAX, the Gavi-led financing scheme to provide COVID-19 vaccines to low-income and middle-income countries (LMICs), planned to have 2 billion vaccine doses available by the end of 2021 [9,10,11]. Despite these advances, the vaccination of two-thirds of Africa’s 1·2 billion population will still require massive investment for programmatic efforts to overcome vaccine hesitancy in this region [10]. According to a WHO analysis, the African region has an average score of 33% readiness for a SARS-CoV-2 vaccine roll-out—far below the necessary 80% benchmark [12]. COVAX aims to secure enough doses of available COVID-19 vaccines to protect an initial 20% of people at most risk in signatory countries [10]. Healthcare workers (HCWs) are a critical high-risk group and play a vital role in advising patients and communities about vaccination [13]. Hence, it is essential to consider their attitudes and risk perceptions about COVID-19 vaccines as these variables mirror their vaccine acceptance. Whereas much information is known regarding vaccine acceptance for developed countries, little is known about COVID-19 vaccine acceptance in Sub-Saharan Africa [14].

Worrisomely, Nigeria as a country has once had a severe case of mass vaccine refusal, an event which took place in the year 2003–2004, when Northern Nigeria boycotted the polio vaccination program, which led to a resurgence of the disease in the country and beyond [15,16,17,18]. To this end, this survey study investigates the acceptance, risk perceptions, attitudes and predictors of COVID-19 vaccine acceptance among HCWs in Nigeria. This is, to the best of our knowledge, the broadest study undertaken to determine COVID-19 vaccine acceptance among HCWs considering the major geopolitical zones in Nigeria.

## 2. Methods

### 2.1. Study Design and Data Collection

A self-administered anonymous manual survey was conducted among HCWs in selected states in all the geographical zones in the country between 28 September and 30 March 2022. The invitation for this survey contained content providing information regarding study purposes, procedures and confidentiality.

### 2.2. Inclusion Criteria

Inclusion criteria included (1) being between 15 and 65 years of age, (2) currently being a healthcare worker in a designated healthcare facility in Nigeria and (3) being willing to supply the information contained in the questionnaire.

### 2.3. Sampling

A descriptive cross-sectional sampling method was utilized. The survey tool was distributed manually to healthcare workers in various healthcare facilities in the selected representative state in the Northern (Taraba State, Abuja), Western (Lagos State, Ondo State), and Eastern (Enugu State, Anambra State) regions.

### 2.4. Power Analysis

We used the Cochran formula (1997), N = $\frac{P\left(1-p\right)Z^{2}}{e^{2}}$, to estimate the sample size due to the fluid population size of HCWs in Nigeria. For our heterogeneous population, we hypothesized a greater variability of 50%; therefore, *p* = 0.5. The margin of error *e* = 0.05, while *Z* = 2.58 at reliability level of 99%. Substituting these values in the equation above, we obtained N = 665.64, although we obtained 710 respondents in our study.

### 2.5. Data Analysis

The data entered were initially vetted for possible data entry error(s), and proper coding of descriptive statistics for sociodemographics, COVID-19 vaccine acceptance, risk perception and general attitudes towards COVID-19 vaccination was performed. Cross tabulations were performed to determine statistical relations between sociodemographic variables, knowledge, attitudes and risk perceptions concerning COVID-19 vaccine acceptance. To provide insights into the association between COVID-19 vaccine acceptance and a variety of independent variables, we categorized the response to the question “Will you take COVID-19 vaccine if made available?” as our result of interest and utilized multinomial logistic regression analysis to determine the predictive variables. Multinomial logistic regression was used because the dependent categories were structured to have three nominal categories (yes, no, not sure). For sociodemographic categories, we varied the reference variable while we chose extreme variables such as “no” and “not sure” for other study categories for easy interpretation and relevance to answering our assumptions on vaccine acceptance. Confounding effects were assessed by observing significant changes in the coefficient after eliminating possible confounding variables. Our final multinomial logistic regression was assessed for the fitness of the model using the Akaike information criterion (AIC). IBM SPSS Statistics 23 was used for all statistical analyses, and a *p*-value less than 0.05 was considered statistically significant at a CI of 95%, with the results presented in tables, bar charts and pie charts.

## 3. Measured Parameters

### 3.1. Sociodemographic of Respondents

The respondents were asked to supply information on their age group, gender, ethnicity, occupational cohort, ward type, healthcare facility, location and geopolitical zone for data analysis. The sociodemographic data were coded as follows: gender (1 = male, 2 = female); ethnicity (1 = Igbo, 2 = Hausa, 3 = Yoruba, 4 = other); occupational cohort (1 = physician, 2 = nurse, 3 = pharmacist, 4 = physiotherapist, 5 = scientist, 6 = radiographer, 7 = administrative staff, 8 = other); ward type (1 = respiratory department, 2 = emergency department, 3 = intensive care unit, 4 = medical imaging, 5 = medical laboratory, 6 = other); location (1 = urban, 2 = suburban, 3 = rural); geopolitical region (Northern = 1, Eastern = 3, Western = 4, other = 5).

### 3.2. Acceptance of COVID-19 Vaccine

To assess participants’ perceptions and willingness to take the COVID-19 vaccine, the following question was asked: Will you take the COVID-19 vaccine if made available (yes, no, not sure)?

### 3.3. Knowledge and Attitudes towards COVID-19 Vaccination

In assessing the participants’ general attitudes towards COVID-19 vaccination and knowledge of the different kinds of vaccines, the following questions were asked: Have you taken any vaccine for the past three years (yes, no, not sure)? Will you recommend the COVID-19 vaccine to your patient (yes, no, not sure)? Do you know the different COVID-19 vaccines currently in use in Nigeria (yes, no, not sure)? Do you trust professional staff advice on the COVID-19 vaccine (yes, no, not sure)? Is the timing for the current COVID-19 vaccine appropriate (yes, no, not sure)? Do you think COVID-19 vaccines should be made mandatory (yes, no, not sure)? What are the most reliable sources of information for the COVID-19 vaccine (NCDC, WHO, pharmaceutical industries, social media, religious bodies, family, friends and others)? Which approved COVID-19 vaccine candidate do you prefer (Pfizer–BioNTech, Moderna, Oxford–AstraZeneca, COVAX BBIBP-CorV, Covaxin, CoronaVac, CoviVac, Sputnik V, Convidicea, Johnson & Johnson, EpiVacCorona, other)?

### 3.4. Risk Perception of COVID-19 Vaccine

Participants’ risk perception of COVID-19 vaccines was assessed by asking them the following questions: Do you have any medical comorbidities (no, yes)? Are you concerned about the side effects of the COVID-19 vaccine (yes, no, not sure)? Do you think the COVID-19 vaccine contains dangerous substances (yes, no, not sure)? Do you think that the current COVID-19 vaccine has passed full clinical trials (yes, no, not sure)? Do you think the COVID-19 vaccine is safe (yes, no, not sure)?

## 4. Results

### 4.1. Descriptive Statistics and Multinomial Logistic Regression of Sociodemographic Characteristics and COVID-19 Vaccine Acceptance among HCWs in Nigeria

#### 4.1.1. Descriptive Statistics

**Figure 1:**
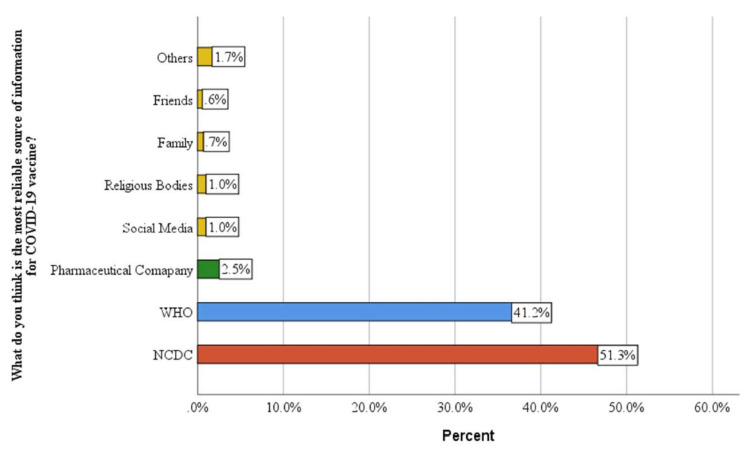
Reliability of COVID-19 sources of information across COVID-19 vaccine landscape.

**Figure 2:**
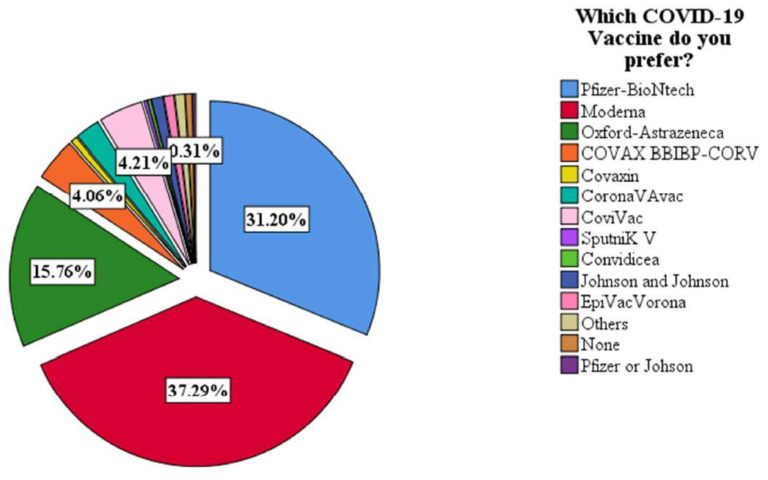
COVID-19 vaccine preference among HCWs.

Seven hundred ten healthcare workers completed the survey, including 281 (39.6%) males and 429 (60.4%) females. The age groups 15–25 years and 26–36 contributed 71.7% of the total respondents. Of the 710 respondents, 307 (43.2%) were from healthcare facilities in Northern Nigeria. A total of 421 (60.0%) of the healthcare facilities were located in urban regions. In terms of ethnicity, 229 (32%) of the respondents were Igbo, 135 (19.2%) were Hausas, 193 (27.4%) were Yorubas and 147 (20.9%) were from other ethnic groups (Table 1). Of all the respondents, 587 (81.9%) had no known chronic illness, while 128 (18.0%) said “yes” they have. The majority of the respondents (51.3%) found the Nigerian Centre for Disease Control (NCDC) to be the most reliable source of data on the COVID-19 vaccine (Figure 1). Equally, the participants had a higher preference for the Moderna and Pfizer vaccines as shown in Figure 2. More details on the sociodemographic characteristics are shown in the first column of Table 1.

#### 4.1.2. COVID-19 Vaccine Acceptance

**Figure 3:**
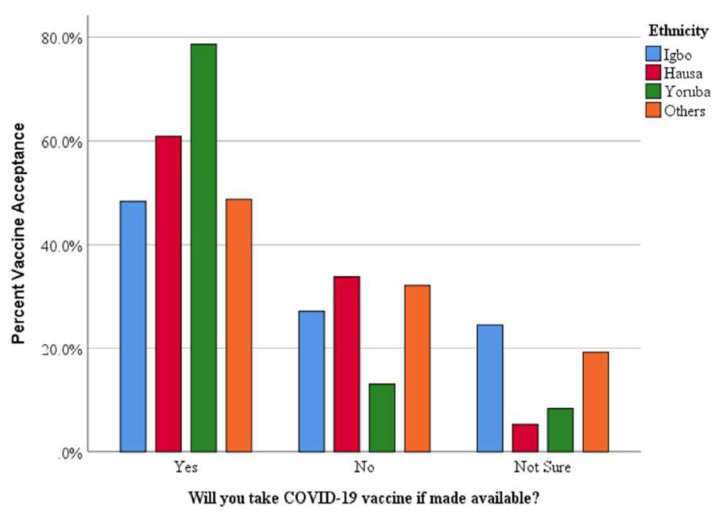
Vaccine acceptance across various ethnicities in Nigeria.

Of the 710 participants surveyed, 418 (59.3%) said they would accept a COVID-19 vaccine (Table 1). Vaccine acceptance among the healthcare population differed by several demographic characteristics: males (69.9%), females (54.1%), Western Nigeria (83.0%), Northern Nigeria (55.1%) and Eastern Nigeria (42.1%). Healthcare workers in facilities located in urban regions had a higher vaccine acceptance of 60.0%. In addition, the Yoruba ethnic group had the highest vaccine acceptance, 78.6% (Figure 3). More details on the vaccine acceptance of the demographics are shown in the third column of Table 1.

#### 4.1.3. Multinomial Logistic Regression

The odds ratios, levels of significance and confidence intervals for sociodemographic variables influencing vaccine acceptance were determined using multinomial logistic regression analysis (Table 1, Column 3). After adjusting for sociodemographic variables, HCWs who were males were 2.8 times more likely to accept the COVD-19 vaccine than females. Other sociodemographic predictor variables that determined vaccine acceptance (at α < 0.05) are healthcare workers in the Northern and Western regions; the Hausa and Yoruba ethnicity; healthcare workers who work in urban healthcare facilities, primary healthcare facilities and private healthcare facilities; and physicians.

### 4.2. Multinomial Logistic Regression of COVID-19 Vaccine Acceptance and Attitudes/Knowledge of Respondents towards COVID-19 Vaccines

As shown in Table 2, 46.6% of the respondents were sure to have taken a vaccine in the past three years and are 3.1 times more likely to take the COVID-19 vaccine than those that were not sure. In addition, 62.3% of the participants would recommend the COVID-19 vaccine to their patients and were 24.9 times more likely to take the COVID-19 vaccine than those that were not sure. Of the participants, 64.8% knew the different COVID-19 vaccines in use and were 1.6 times more likely to subject themselves to vaccination than those that were not sure. Again, 53.6% of the participants agreed that the timing of the COVID-19 vaccination was appropriate and were 4.4 times more likely to subject themselves to being vaccinated than those that were not sure. Similarly, 32.3% of the participant believed that the COVID-19 vaccine should be made mandatory and were 7.7 times more likely to take the vaccine than those that were not sure (Table 2).

### 4.3. Multinomial Logistic Regression of COVID-19 Vaccine Acceptance across Risk Perception Characteristics

As shown in Table 3, 81.3% of the respondents were concerned about the side effects of COVID-19 vaccines, and 51.6% thought that the current COVID-19 vaccine has passed complete clinical trials. In addition, 33% of the respondents believed that the COVID-19 vaccines contain dangerous substances, and 47.7% agreed that the vaccines are not safe. Furthermore, participants that believed that the COVID-19 vaccine has passed complete clinical trials were 6.1 times more likely to take the COVID-19 vaccine than those that were not sure. Those that thought that the vaccine is safe and does not contain dangerous substances were 5.2 and 4.5 times, respectively, more likely to take the COVID-19 vaccine than those that were not sure (Table 3).

## 5. Discussion

The modality for COVID-19 vaccine distribution in low- and middle-income countries follows a hierarchy of necessity based on subgroups with the highest risk exposure due to the lack of enough COVID-19 vaccines to reach everybody. HCWs are among the priority group to have access to these vaccines in Nigeria and are likely to be an essential source of inspiration for COVID-19 vaccine uptake among the general population [13]. Several studies have been conducted to unravel vaccine acceptance patterns particularly among the general population, obviating focus groups such as HCWs [19,20]. It is therefore pivotal to provide insights on the factors associated with vaccine acceptance among this sub-group to provide data for a precise programmatic effort to expedite vaccine uptake among HCWs in Nigeria.

To the best of our knowledge, this is one of Nigeria’s most comprehensive studies on the adoption of the COVID-19 vaccine among healthcare professionals in terms of the integration of the main geopolitical zones. Vaccination acceptance rates varied by age, ethnicity, zones, cohorts, healthcare facilities and other non-sociodemographic characteristics in our study, which included 710 healthcare professionals. These results generally highlight the significance of specialized communication strategies to spread scientific information and boost HCWs’ trust in the COVID-19 vaccine.

The majority of respondents in this study (43.4%) were under the age of 25, which is consistent with many studies conducted in low- and middle-income countries [13,21,22] but parallels those conducted in developed countries [19,23,24]. Higher representation of younger demographics may be explained by the exponential growth of Nigeria’s young population, lower life expectancy, the education law that allows students as young as 16 to pursue any medical or medically related profession in any public institution and the preference for hiring younger graduates [25,26]. However, in contrast to our finding, most studies in other regions of the world found the mean age of respondents to be above 25 years [21,27]. This may have an impact on how generalizable our findings are, especially considering that many studies on the acceptance of the COVID-19 vaccine have found that being an adult older than 30 years of age is predictive of COVID-19 vaccine acceptance [18,27,28,29].

In addition, the majority of respondents (60.4%) were female, and the three major tribes in Nigeria participated in this survey in the following order: Igbo (229; 32.5%), Yoruba (193; 27.4%) and Hausa (135; 19.2%). This study’s larger proportion of female participants is consistent with the findings of prior studies [13,19,30]. In contrast, fewer women responded in some other trials [31,32,33]. The majority of participants (59.3%) were open to receiving the COVID-19 vaccine, with the youngest age group, 37–47, having the highest vaccine acceptance (68.3%), and the oldest, 59–69, having the lowest (55.1%). Although the study carried out by Agha et al. [31] also showed a higher vaccine acceptance of 68.5% in a similar age range of 30–39, there is a broader consensus that vaccine acceptance is higher in the older age group [1,19,27]. 

In this study, the Western region and the Yoruba ethnicity were identified to have the highest vaccine acceptance of 81.3% and 78.6%, respectively, among Nigeria’s three major regions and ethnicities. The higher vaccine acceptance rate is probably because Western Nigeria, precisely Lagos State, is the epicenter of the pandemic in Nigeria, and a plethora of studies have shown that the higher the perceived risk of COVID-19 is, the greater the vaccine acceptance is [19,27,34,35]. 

As shown in Table 1, the trend of the perceived risk of COVID-19 infection mirroring vaccine acceptance [24] was also observed in other demographics, with cohorts involved in direct patient care and those working in the intensive care unit having higher vaccine acceptance of $\bar{x}$ = 59.8% and 67%, respectively, in their categories. According to Yilma et al. [36], motivation to receive vaccines is positively influenced by one’s level of exposure and enlightenment. This may account for our study’s findings that healthcare personnel in urban areas are more likely to accept vaccinations than those in the suburbs.

Additionally, a survey of 1182 HCWs in Singapore who only worked in primary healthcare institutions found that 95% of them were in favor of vaccinations [37]. Consistent with this outcome, the primary healthcare workers in our study had the greatest vaccination acceptance rate (83.5%) when compared to HCWs in other facilities.

The trust of HCWs in national institutions will have a direct relationship with their level of acceptance of information they disseminate, affecting their compliance with government directives. Thus, in response to the question regarding the most reliable source of data on the COVID-19 vaccine, the majority of the respondents (about 51.3%) preferred the Nigeria Centre for Disease Control (NCDC), while 41.2% chose the World Health Organization, as shown in Figure 1. The relatively heavy reliance on NCDC and WHO for information about COVID-19 vaccines may be attributed to the quality and reliability of the information published weekly, monthly and quarterly by the institutions on current issues about COVID-19 vaccines, especially on the safety profile, clinical trials and approval. Similarly, a study among Indians revealed that a little less than two-thirds (64.5%) of the respondents have higher trust in India’s healthcare system [30]. 

While the US FDA halted the use of Johnson & Johnson COVID-19 vaccinations following numerous suspected and documented deaths and serious cases of a form of blood clotting following immunization, several European countries suspended the AstraZeneca COVID-19 vaccine in March and April 2021 [38,39]. These incidents would unquestionably raise the perceived risks associated with potential vaccination [40]. This may explain the preference for the Moderna and Pfizer COVID-19 vaccines (as shown in Figure 2) relative to the AstraZeneca COVID-19 vaccine which was better made available by the COVAX, Gavi-led vaccination support in Nigeria [41].

Additionally, the multinomial logistic regression analysis was used to assess the determinants of COVID-19 vaccine acceptance, and it was discovered that female HCWs were 2.8 times less likely to receive the vaccination than male HCWs. This outcome is in line with findings from other studies [24,42,43]. There is a favorable association between strong vaccine acceptance and risk perception, according to earlier studies [42,43,44,45,46]. As males have been found to have more COVID-19-related morbidity and mortality, this may explain why they are more likely than females to accept the COVID-19 vaccine. Additionally, there is a dearth of information regarding how COVID-19 vaccines affect expectant mothers, which may contribute to a poor perception of risk [44,45,46].

In contrast to the Igbo and other minor ethnic groups, the Yoruba and Hausa ethnicities were shown to have increased vaccine acceptance rates. Given that the Western and Northern regions were predominantly the pandemic’s epicenter in Nigeria, this is likely because of the higher perceived risk of COVID-19 vaccine infection in those areas. In a similar pattern, a study carried out in four states in the USA demonstrated that states that were badly hit by the pandemic had a lower vaccine hesitancy rate [47]. The Yoruba ethnic group had the highest vaccine acceptance in this study, with a score of 78.6%, as shown in Figure 3. Additionally, a study that sampled 440 respondents in Ibadan, a western state of Nigeria, reported a high positive vaccine acceptance of 79.5% [48], similar to the vaccine acceptance in this study.

Furthermore, the study carried out in Southern California and Ethiopia among HCWs reveals that safety concerns and educational status played a huge role in the higher vaccine acceptance in physicians compared to other HCWs [23,36]. This present study similarly identified physicians and HCWs in the intensive care unit as predictors of COVID-19 vaccine acceptance. Finally, among other sociodemographic variables, HCWs in primary and private healthcare facilities contributed to vaccine acceptance, probably due to the higher vaccine acceptance observed in the descriptive statistics (Table 1, Column 1).

Regarding risk perception and attitudes toward vaccination with vaccine acceptance, the regression analysis further indicated seven factors (bordering safety and positive attitude towards vaccination) significantly associated with the willingness to accept COVID-19 vaccines (Table 2 and Table 3). These factors were corroborated in a multicenter study of 1398 HCWs in 20 emergency departments in the United States, where the main reason for declining the COVID-19 vaccine was linked to vaccine safety [27]. The results of this study showed that HCWs who believe that the vaccines have passed complete clinical trials and are safe are 6.1 and 5.2 times more likely to accept the vaccine than those who are not sure. Many COVID-19 vaccine hesitancy studies worldwide have identified similar concerns among HCWs and the general population [49,50,51]. 

Why HCWs do not think the COVID-19 vaccines have passed through complete clinical testing [24] may be explained by the COVID-19 vaccines’ record development in less than a year. Additionally, 33% of participants believed the COVID-19 vaccination contained a dangerous substance, but those who responded “no” were 4.5 times more likely to receive the vaccine. In a survey conducted before the COVID-19 vaccine was introduced in the nation, Nigerians strongly supported the conspiracy hypothesis that the vaccines contained microchips and were depopulation products [52].

Numerous studies have demonstrated a significant correlation between vaccine acceptance and awareness about and favorable attitudes toward vaccination [13,31,53]. These results are supported by our regression analysis, which shows that participants who are willing to recommend the vaccine to their patients and have received other vaccinations within the previous three years are 3.1 and 24.9 times more likely to receive a COVID-19 vaccine than participants who are unsure about having received other vaccinations within the previous three years and are unsure about recommending the vaccine.

In contrast to a study [27] that found that vaccination acceptability is inversely correlated with willingness to see others receive the vaccine, our investigation found an association between readiness to suggest COVID-19 shots and vaccine acceptance. Finally, participants who are aware of the several COVID-19 vaccines readily available in Nigeria and who believe that the time is right to get vaccinated are 1.6 times and 4.4 times more likely to receive the vaccines, respectively. Education regarding vaccines is strongly correlated with vaccine acceptability, according to numerous studies [20,54,55]. Therefore, vaccine acceptability is higher the more information a person has about the COVID-19 vaccines that are accessible.

## 6. Conclusions

Only 59.3% of the participants were amenable to COVID-19 vaccinations, with some variations across the demographic variables. The main predictors for COVID-19 vaccine acceptance were bordered around safety concerns and potential side effects of the COVID-19 vaccines. Moreover, participants with positive attitudes towards vaccination showed higher vaccine acceptance with a general preference for the Moderna and Pfizer vaccines. Our findings also highlighted the suitability of the Nigeria Centre for Disease Control, among other information sources, to frame public health messages to best address vaccine hesitancy among HCWs in Nigeria. Resultantly, COVID-19 campaigns should be tailored to specific subgroups to enhance the understanding of the science underlying vaccine clinical trials. Interventions should be addressed sensitively, and subgroups who are reluctant to receive vaccines should be taken into account when establishing policies.

## Figures and Tables

**Table 1:** Demographic characteristics and multinomial logistic regression for COVID-19 vaccine acceptance among HCWs in Nigeria.

Variables	n (%)	COVID-19 Vaccine Acceptance (Total = 418/705; 59.3% n (%))	OR	*p*-Value	95% CI
Gender					
Male	281 (39.6%)	188 (66.9%)	2.8	<0.001	1.7–4.6
Female	429 (60.4%)	232 (54.1%)	Ref		
Age					
15–25	308 (43.4%)	163 (52.8%)	0.6	0.5	0.1–2.9
26–36	202 (28.5%)	126 (62.4%)	0.8	0.8	0.7–4.0
37–47	102(14.3%)	70 (68.6%)	0.7	0.6	0.1–3.3
48–58	75 (10.5%)	42 (65.3%)	0.8	0.8	0.1–4.4
59–69	22 (3.1%)	13 (59.1%)	Ref		
Zone in Nigeria					
North	307(43.2%)	169 (55.1%)	0.4	0.004	0.2–0.8
East	202 (28.5%)	85 (42.1%)	0.1	<0.001	0.1– 0.3
West	201 (28.3%)	167 (83.0%)	Ref		
Ethnicity					
Igbo	229 (32.5%)	110 (48.2%)	0.8	0.357	0.5–1.3
Hausa	135 (19.2%)	82 (60.9%)	4.5	<0.001	1.8–10.9
Yoruba	193 (27.4%)	152 (78.6%)	3.5	<0.001	1.8–7.1
Others	147 (20.9%)	71 (48.6%)	Ref		
Location					
Urban	421 (60.0%)	269 (63.9%)	2.4	0.004	1.5–4.8
Suburban	192 (27.4%)	98 (51.1%)	1.1	0.7	0.3–1.4
Rural	89 (12.7%)	48 (53.9%)	Ref		
Facility					
Teaching Hospital	119 (17.0%)	55.9 (47.0%)	0.364	1.442	0.7–3.2
Federal Medical Centre	228(32.5%)	132 (57.9%)	0.083	1.733	0.9–3.2
Private Hospital	138 (19.7%)	91 (65.7%)	1.7	<0.020	0.8–3.7
Primary Healthcare	103 (14.7%)	86 (83.2%)	4.4	<0.004	1.6–12.3
Others	114 (16.2%)	52 (45.6%)	Ref		
Ward					
Respiratory Unit	53 (7.6%)	29 (54.7%)	2.9	0.1	0.6–3.8
Emergency Unit	104 (14.9%)	67 (64.4%)	2.0	0.17	0.3–2.0
Intensive Unit	106(15.2%)	71 (67.0%)	2.0	0.18	0.3–2.0
Medical Imaging	28 (4.0)	19 (66.7%)	2.5	0.2	0.2–2.4
Medical Laboratory Unit	41 (5.9%)	23 (56.5%)	1.4	0.5	0.5–2.3
Others	367 (52.5%)	197 (53.7%)	Ref		
Cohort					
Physician	111(15.7%)	69 (62.4%)	2.4	0.031	1.1–5.4
Nurse	46(6.5%)	29 (62.2%)	2.0	0.9	0.4–2.2
Pharmacist	157(22.2%)	101 (64.1%)	1.2	0.5	0.7–2.2
Physiotherapist	20(2.8%)	15 (75.0%)	4.8	0.1	0.6–37.8
Scientist	114(16.1%)	50 (43.9%)	0.6	0.2	0.3–1.2
Radiographer	27(3.8%)	21 (76.9%)	3.2	0.1	0.7–14.4
Administrative Staff	50(7.0%)	35 (70.0%)	2.8	0.7	0.9–8.5
Others	181 (25.5%)	100 (55.2%)	Ref		
Chronic Illness					
Yes	128 (18.0%)	79 (61.4%)	1.1	0.8	0.670–1.665
No	578(81.9%)	339 (58.7%)	Ref		

**Table 2:** Multinomial logistic regression of COVID-19 vaccine acceptance and attitudes/knowledge of respondents towards COVID-19 vaccines.

Knowledge and Attitude towards COVID-19 Vaccination		n %	COVID-19 Vaccine Acceptance	OR	*p*-Value	95% CI
Have you taken any other vaccine apart from the COVID-19 vaccine in the past three years?	Yes No NS	328 (46.7%) 330 (46.9%) 45(6.4%)	235 71.6%) 162 (49.1%) 19 (42.2%)	3.1 Ref.	0.027	1.1–8.3
Will you recommend the COVID-19 vaccine to your patient?	Yes Not NS	438 (62.8%) 155(22.2%) 105 (15.0%)	364 (83.1%) 30 (19.4%) 18 (17.1%)	24.9 Ref.	<0.001	12.6–49.3
Do you know the different COVID-19 vaccines currently in use in Nigeria?	Yes No NS	458 (66.4%) 157 (22.8%) 75 (10.9%)	315 (68.8%) 59 (45.7%) 35 (8.6%)	1.6 Ref.	0.024	0.5–5.2
Is the timing for the current COVID-19 vaccine appropriate	Yes No NS	378 (53.8%) 120 (17.1%) 204 (29.1)	299 (79.1) 52 (43.3%) 66 (32.4%)	4.4 Ref	<0.001	2.4– 8.0
Do you think COVID-19 vaccines should be made mandatory?	Yes No NS	224 (32.3%) 376 (54.2%) 94 (13.5%)	203 (90.6%) 151(40.3%) 56 (59.6%)	7.6 Ref.	<0.001	3.3–17.8

NS = not sure = reference variables.

**Table 3:** Multinomial logistic regression of COVID-19 vaccine acceptance across risk perception characteristics.

Risk Perceptions and COVID-19 Vaccination		n%	COVID-19 Vaccine Acceptance	OR	*p*-Value	95% CI
Are you concerned about the side effects of COVID-19 vaccines?	Yes No	569 (81.2%) 132 (18.8%)	348 (61.2%) 67 (50.8%)	1.5 Ref	0.3	0.8- 2.8
Do you think the current COVID-19 vaccines have passed full clinical trials?	Yes No NS	364 (51.8%) 206 (29.5%) 133 (18.9%)	317 (87.1%) 57 (27.7%) 43 (32.3%)	6.1 1.4 Ref	<0.001 0.3	3.1–12.2 0.7–2.6
Do you think the COVID-19 vaccine contains dangerous substances?	Yes No NS	229 (33%) 253 (36.2%) 694 (30.8%)	123 (53.7%) 206 (81.4%) 409 (58.9%)	1.7 4.5 Ref	0.106 <0.001	0.9–3.1 2.0–10.1
Do you think COVID-19 vaccine is safe?	Yes No NS	337 (47.9%) 138 (19.5) 228 (32.8%)	305 (90.5%) 40 (29.0%) 72 (31.6%)	5.2 0.7 Ref	<0.001 0.8	2.50–10.92 0.4–1.74

NS = not sure = reference variable.

## Data Availability

Not applicable.
